# Parental perception of FIRES outcomes, emotional states, and social media usage

**DOI:** 10.1002/epi4.12513

**Published:** 2021-06-21

**Authors:** Raquel Farias‐Moeller, Alexandra Wood, Rachel Sawdy, Jennifer Koop, Krisjon Olson, Andreas van Baalen

**Affiliations:** ^1^ Division of Child Neurology Department of Neurology Medical College of Wisconsin Children's Wisconsin Milwaukee WI USA; ^2^ Division of Critical Care Department of Pediatrics Medical College of Wisconsin Children's Wisconsin Milwaukee WI USA; ^3^ Division of Pediatric Neuropsychology Department of Neurology Medical College of Wisconsin Children's Wisconsin Milwaukee WI USA; ^4^ Department of Neuropediatrics University Medical Center Schleswig‐Holstein Christian‐Albrechts‐Universität zu Kiel Kiel Germany

**Keywords:** epilepsy, febrile infection‐related epilepsy syndrome, new‐onset refractory status epilepticus, social media

## Abstract

**Objective:**

To investigate parental perception of FIRES outcomes, assess emotional states and related social media usage.

**Methods:**

A survey‐based study of parents of children with FIRES participating in a FIRES‐specific Facebook group was performed. The survey collected information on medical aspects of their child's course in the acute, subacute, and chronic periods, emotional states, and social media usage. Child outcome was assessed utilizing the pediatric extended Glasgow outcome scale (GOS‐E). Parental emotional states were assessed utilizing the Depression, Anxiety and Stress Scale (DASS). Descriptive statistics were performed. Associations were described using the Spearman rank correlation. Open‐ended questions were included. Thematic analysis was performed.

**Results:**

Twenty‐nine surveys were analyzed. All children were in the chronic phase at time of survey response, except for two who died. Mothers answered 22 surveys, and fathers answered seven. Median age at FIRES presentation was 5.6 years [IQR 4.2‐8.95], with a median number of 3 seizures per week [IQR 0‐10, range 0‐50], 4 daily anti‐seizure medicines [IQR 3‐5], and chronic GOS‐E of 6 [IQR 2‐8 range 2‐8]. Most parents reported none to mild levels of depression, anxiety, and stress. Higher seizure burden positively correlated with parental depression symptoms (*r* = .41 (95% CI 0.01, 0.70), *P* = .045). Most parents found social media helpful with coping and 96% desired FIRES research advertised. Twenty‐five parents shared their recommendations to fellow parents and the medical team in an open‐ended format. Themes included support, expertise, and medical advice.

**Significance:**

Despite their children's significantly impaired functional outcome after FIRES and high rates of medically refractory epilepsy, the cohort demonstrated remarkable emotional resilience. They perceive social media as beneficial, are interested in social media‐advertised research, and share valuable advice. Social media may serve as an introductory platform to enhance the physician‐scientist‐parent/patient relationship.

**Key Points**

- This is a survey of parents of children with FIRES who participate in FIRES‐specific social media, assessing child's outcome, parent's emotional states, and social media usage.
- Child outcomes were poor. Median weekly seizures, daily anti‐seizure medicines, and extended Glasgow outcome scales were 3, 4, and 6, respectively.
- Most parents reported none‐mild levels of depression, anxiety, and stress. Higher seizure burden positively correlated with parental depression symptoms.
- Most parents found social media helpful with coping and 96% desired FIRES research advertised.
- Parents shared recommendations to other parents and medical team. Themes included support, expertise, and medical advice.

## 1 INTRODUCTION

New‐onset refractory status epilepticus (NORSE) is a very rare clinical presentation of refractory status epilepticus (RSE) without obvious cause in a patient without pre‐existing disease. Febrile infection‐related epilepsy syndrome (FIRES), a subcategory of NORSE, is characterized by a febrile illness 2 weeks to 24 hours preceding RSE.^1^ Although the diagnosis of NORSE or FIRES can be given at any age, FIRES is more commonly seen in typically developing school‐aged children.^2^ The prodromal illness is usually mild, and shortly after the first seizure, an explosive RSE ensues. Up to hundreds of daily seizures dominate the acute period and without quiescence, the chronic epileptic encephalopathy follows. The pathophysiology remains elusive though intrathecal inflammation, and activation of innate immune mechanisms is thought to play a role.^2^

Febrile infection‐related epilepsy syndrome is an incredibly difficult condition to manage for clinicians, due to its rarity, lack of diagnostic criteria, seizure severity, elusive etiology, and significant morbidity. Counseling families in this setting is uniquely challenging due to multiple factors like delays in diagnosis, lack of an etiology or diagnostic markers, and relative uncertainty of spectrum of outcome.^3^ For most families, this unheralded condition is devastating. Death occurs in 12%, return to pre‐morbid states is rare, and survivors often experience severe encephalopathy with high rates of medically refractory epilepsy.^4^, ^5^

Families dealing with rare diseases are active in social media communities,^6^ and families of patients with FIRES are no exception. Facebook is a large social media platform with 2.7 billion monthly active users and 1.79 million daily active users in 2020.^7^ Facebook engagement has been used successfully to study pediatric rare diseases.^8^ The FIRES Facebook group was created in September 2012 by a parent of a child with FIRES. The mission of the group is to connect families dealing with this rare disease. The group is private, and information is proctored by a group of parent administrators. As of December 2020, it had 758 members.^9^

We hypothesized that families dealing with FIRES and active in social media would be willing to participate in a descriptive study. Our survey‐based approach aimed to investigate parental perception of FIRES outcomes, assess emotional states and social media usage.

## 2 METHODS

### 2.1 Study design and survey characteristics

The study was approved by the Medical College of Wisconsin (PRO00036742) Institutional Review Board. A conditional branching survey was developed by the study authors: a pediatric epileptologist (RFM), a pediatric neuropsychologist (JK), and a medical anthropologist (KO). The survey consisted of six sections: demographics, pre‐FIRES presentation, acute FIRES stage, acute FIRES complications, subacute/chronic FIRES section and emotional states, and social media usage questions. Emotional states were assessed utilizing the 42‐point Depression, Anxiety and Stress Scale (DASS), and responses were individually rated according to questionnaire instructions.^10^ Child outcome was assessed using the pediatric extended Glasgow outcome scale.^11^ Impact of the Facebook group on coping and parenting was assessed utilizing a 1‐10 Likert scale where 1 = not helpful, 10 = most helpful. Open‐ended questions were used to solicit parent participant recommendations for families dealing with a child with FIRES and recommendations for the medical team dealing with a family of a child with FIRES. (Appendix S1).

### 2.2 Survey recipients and distribution

The administrators of the social media group “FIRES” were asked to participate in a questionnaire that collected basic demographic data of the social media group. The administrators were then asked to post an advertisement directed at family members of children with FIRES. If interested, family members were instructed to contact an email address that contained an automatic reply with a unique web address directing them to the survey. Surveys of family members of previously typically developing children with a history of refractory status epilepticus (RSE) without a known cause and preceded by fever were included in the analysis. We excluded surveys of children younger than 2 years (to avoid inclusion of infantile‐onset epileptic encephalopathies) or with pre‐existent epilepsy, and those who were >50% devoid of answers.

The survey was generated using Qualtrics software, version XM of Qualtrics. The estimated time for survey completion was 45 minutes. Families were advised of the sensitive nature of questions and encouraged to skip ahead if they desired. The families could return to the survey if they needed to pause and resume later. The original advertisement was posted in May of 2020 and re‐posted until survey closure date of September 2020. Members and administrators re‐posted the advertisement every 2‐3 weeks. The survey was generated using Qualtrics software, version XM of Qualtrics.

### 2.3 Survey analysis

Descriptive statistics were performed according to rate of response of respondents (not all respondents answered all questions). Associations were described using the Spearman rank correlation. The analysis was performed using SAS version 9.4 (SAS Institute). Open‐ended questions were analyzed through coding. The open coding method consisted of sorting responses into groups based on shared themes. The axial coding method categorized all codes subsequently into broader themes. For example, a comment providing specific recommendations on phenobarbital dosing would be open coded as “therapeutics”, and the axial code under which it would fall would be “providing specific medical advice”. We analyzed all open‐ended questions and extracted themes until saturation was reached, that is no new themes emerged. Discrepancies were discussed with the authors until consensus was reached.

## 3 RESULTS

**FIGURE 1:**
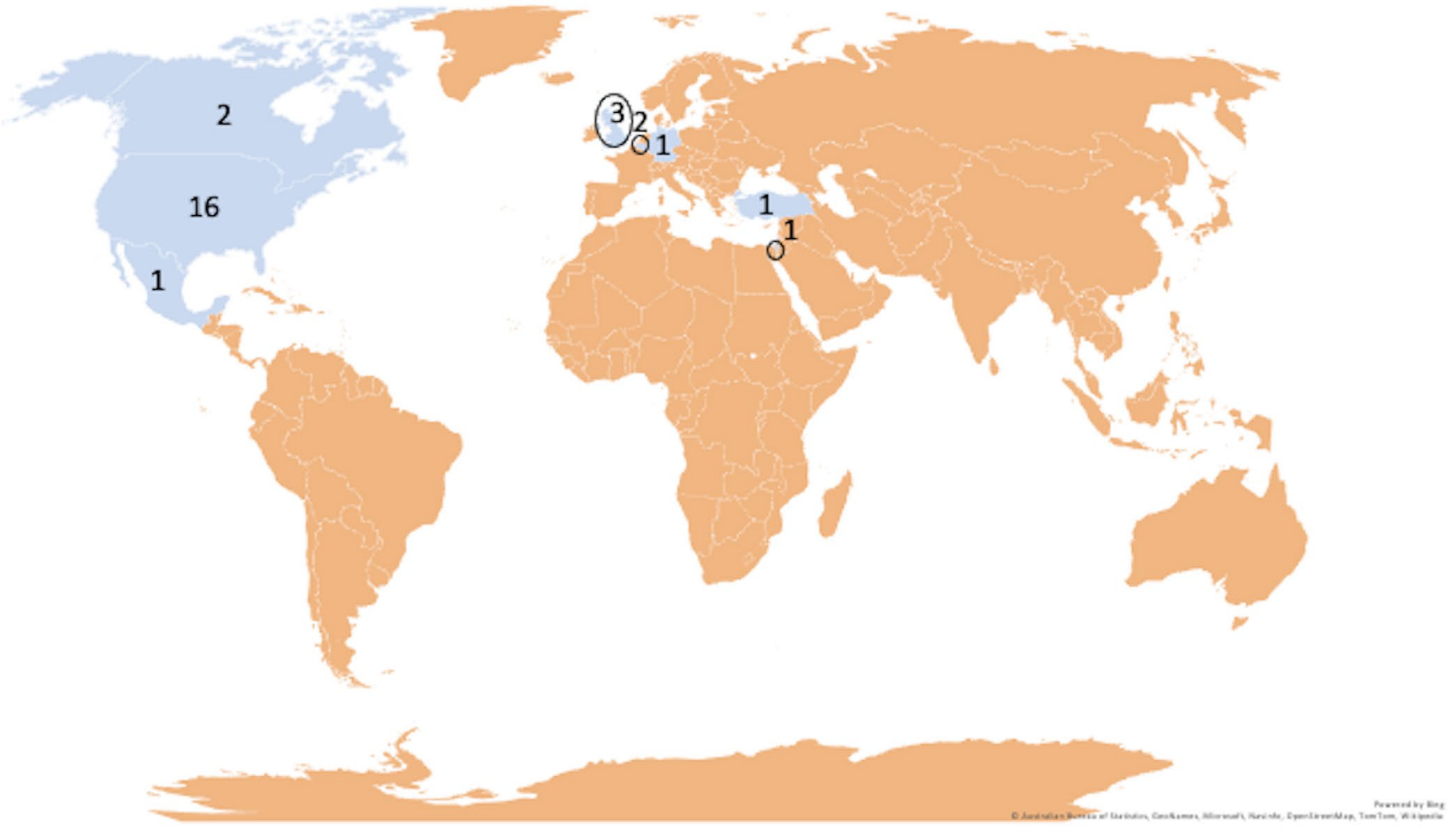
Location of patients when they were first diagnosed with Febrile infection‐related epilepsy syndrome (FIRES)

We received 47 requests for surveys via email. Thirty‐three surveys were completed. Four were excluded due to: age <2 years (n = 1) and completely devoid of answers (n = 3). Twenty‐nine surveys were analyzed. This was an international sample, and respondents were from various locations (Figure 1).

### 3.1 Demographics and characteristics of the acute period

Table 1 describes characteristics of the cohort and the acute FIRES period. The cohort included 6 female children, and mothers were the respondents in the majority of surveys. The median age at FIRES presentation was 5.6 years [IQR 4.2‐8.95]. At the time of survey response, children had a median age of 10.4 yrs [IQR 7.6‐13.7], indicating that the median duration of illness was close to 5 years. The most common prodromal illness was gastrointestinal followed by upper respiratory.

**TABLE 1:** Demographics and characteristics of acute and chronic FIRES period

	n/%
Female gender	6/20.6
Death at time of survey completion	2/6.8
Prodromal illness^a^	
Gastrointestinal	17/58.6
Upper respiratory	8/27.6
Lower respiratory	4/13.8
Rash	4/13.8
Other	8/27.6
Family member filling out survey	
Mother	22/75.9
Father	7/24.1
Median age at time of survey completion	10.4 y [IQR 7.6‐13.7]
Median age at time of FIRES presentation	5.6 y [IQR 4.2‐8.95]
Acute FIRES period	
MIC (n = 29)	26/89.7
Endotracheal intubation (n = 29)	27/93.0
Discharge disposition (n = 27)	
Home	16/59.3
Death	1/3.7
Rehabilitation	10/37.0
Tracheostomy (n = 26)	10/38.5
Gastrostomy (n = 26)	16/61.5
Complications^a^ (n = 29)	
Paroxysmal sympathetic hyperactivity	10/34.5
Skin rash	9/31.0
Intestinal	8/27.6
Deep vein thrombosis	4/13.8
Cardiac arrest	3/10.3
Sepsis	2/6.9
None listed	7/24.1
Median duration of MIC (n = 23)	24 days [IQR 8‐40]
Median duration of intubation (n = 12)	32.5 days [IQR 11.25‐46.75]
Median duration of hospitalization (n = 28)	72 days [IQR 30.5‐99]
Median GOS‐E at time of hospital discharge (n = 26)	6 [IQR 5‐7, range 3‐8]
Chronic FIRES period	
Government assistance/public insurance (n = 26)	18/26.2
Home nursing support (n = 26)	5/19.2
Receives therapies (n = 26)	18/69
Median number of weekly seizures (n = 23)	3 [IQR 0‐10, range 0‐50]
Median number of anti‐seizure medicines (n = 26)	4 [IQR 3‐5]
Median number of neurologists seen (n = 25)	3 [IQR 2‐4]
Median GOS‐E at time of survey completion (n = 26)	6 [IQR 5‐7, range 2‐8]

Note

(n=) indicates # of respondents who answered that question.

Abbreviations: GOS‐E, Extended Glasgow Outcome Scale; MIC, medically induced coma.

^a^ Multiple answers permitted.

Medically induced coma and its duration were used as a surrogate to assess severity of FIRES case. Twenty‐six children (89.7%) were placed in a medically induced coma to control seizures for a median duration of 24 days [IQR 8‐40]. Twenty‐seven (93%) were endotracheally intubated for a median duration of 32.5 days [IQR 11.25‐46.75]. Acute complications were reported by the majority (74%), the most common being paroxysmal sympathetic hyperactivity (PSH), followed by rash. Two children died, one in the acute phase after 21 days, and one in the chronic phase 3 years after presenting with FIRES. Of the twenty‐eight who survived until discharge, 59% were discharged home and 37% to a rehabilitation center. Tracheostomy and gastrostomy were placed in 38.5% and 62%, respectively. The median GOS‐E reported at time of hospital discharge was 6 (lower severe disability) [IQR 5‐7 range 3‐8].

### 3.2 Characteristics of the chronic FIRES period

**FIGURE 2:**
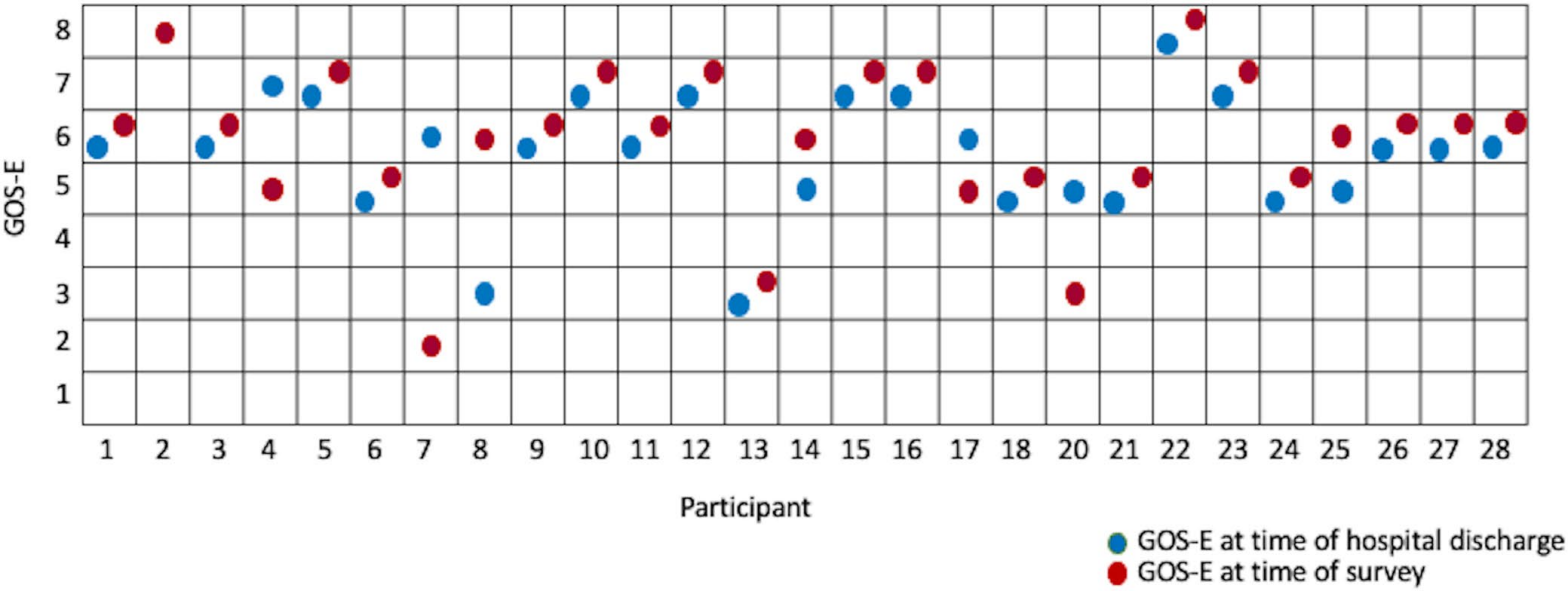
Extended Glasgow Outcome Scale (GOS‐E) for Pediatrics for 28 participants. 1—Upper Good Recovery; 2—Lower Good Recovery; 3—Upper moderate Disability; 4—Lower Moderate Disability; 5—Upper Severe Disability; 6—Lower Severe Disability; 7—Vegetative State; and 8—Death. Blue circle demonstrates GOS‐E at time of hospital discharge after FIRES, and red circle demonstrates GOS‐E at the time of survey. Median interval between hospital discharge and timing of survey was 4.8 years

Twenty‐eight children survived to the chronic FIRES period. In the chronic phase, parents reported a median number of 3 seizures per week [IQR 0‐10, range 0‐50]. Their children were on a median number of 4 anti‐seizure medicines [IQR 3‐5], and one child had a vagus nerve stimulator. They had seen a median number of 3 neurologists [IQR 2‐4]. Rates of government‐issued insurance and home nursing support were low at 26% and 19%, respectively. Sixty‐nine percent required some form of therapies including physical, occupational, or speech therapy. The median GOS‐E reported at the time of survey completion was 6 (lower severe disability) [IQR 2‐8 range 2‐8]. Figure 2 demonstrates outcome variations from the time of discharge to the time of survey completion for 26 children according to their parents. Nineteen children (73%) had no variation across time in GOS‐E score. Four children (15%) improved in functional outcome and three children (11.5%) worsened, including one who died.

### 3.3 Emotional states and social media

Table 2 demonstrates numerical ranges of depression, anxiety, and stress symptoms and categorizes according to normal, mild, moderate, severe, and extremely severe according to the DASS.^10^ Most parents reported none to mild levels of depression, anxiety, and stress (62%, 75.8%, and 72.4%, respectively). Median depression, anxiety, and stress scores were 8 (IQR 2‐19; [normal 0‐9]), 4 (IQR 0‐10; [normal 0‐7]), and 11 (IQR 5‐19; [normal 0‐14]), respectively. Group comparisons (mother vs father respondent) were not performed given overall small cohort and disproportionate low number of fathers who responded. The only variable correlating with higher rate of self‐reporting of depression symptoms was a higher weekly seizure burden (*r* = .41 (95% CI 0.01, 0.70), *P* = .045). A higher number of weekly seizures was not associated with increased stress or anxiety symptoms (*P* = .112, .075, respectively).

**TABLE 2:** Parental coping and social media usage

Median DASS depression score (n = 29)		8 [IQR 2‐19, range 0‐31]
Normal (0‐9)	n/%	15/51.7
Mild (10‐13)		3/10.3
Moderate (14‐20)		7/24.1
Severe (21‐27)		2/22.2
Extremely severe (>27)		2/22.2
Median DASS anxiety score (n = 29)		4 [IQR 0‐10, range 0‐21]
Normal (0‐7)	n/%	17/58.6
Mild (8‐9)		5/17.2
Moderate (10‐14)		4/13.8
Severe (15‐19)		2/6.9
Extremely severe (>19)		1/11.1
Median DASS stress score (n = 29)		11 [IQR 5‐19, range 0‐39]
Normal (0‐14)	n/%	17/58.6
Mild (15‐18)		4/13.8
Moderate (19‐25)		6/20.7
Severe (26‐33)		1/11.1
Extremely severe (>33)		1/11.1
Median impact of FIRES group with coping^a^ (n = 26)		7 [IQR 5‐9.25, range 1‐10]
Median impact of FIRES group with parenting^a^ (n = 22)		5.5 [IQR 2.75‐8.25, range 1‐10]
Desire for physician participation in FIRES group (n = 27)		n/%
Yes		18/66.7
No		2/7.4
Unsure		7/25.9
Ways the FIRES group has changed care for child^b^ (n = 27)		
It has not changed		13/48.1
Started or stopped a medication/diet		7/25.9
Redirected goals of care		4/14.8
Obtained a new test		5/18.5
Joined a research study		7/25.9
Changed physicians		2/7.4
Other		3/11.1
Ways the FIRES group has changed coping^b^ (n = 24)		
It has not changed		3/12.5
I've socialized in person with members		3/12.5
I feel like I help other families		11/41.7
I feel like other families help me		12/50.0
My stress levels are lower		4/16.7
My stress levels are higher		1/4.2
I was more hopeful for the future		5/20.8
I was less hopeful for the future		0/0.0
I was more acceptant of reality		13/54.1
Other		3/12.5

^a^ 1 = not helpful, 10 = most helpful.

^b^ Answers not mutually exclusive.

Approximately 67% of respondents expressed a desire for their physician to join the FIRES Facebook group. On a scale of 1‐10 (1 being least impactful, 10 being the most impactful), the median impact of the FIRES Facebook group on coping and parenting was 7 [IQR 5‐9.25] and 5.5 [IQR 2.75‐8.25], respectively. When asked specific questions on how the FIRES Facebook group helped with caring for their child with FIRES (see Appendix S1 for response options), the most chosen answer was “*It has not changed”,* followed by “*joined a research study*” and “*started or stopped a medication/diet.”* When asked specific questions on how the FIRES Facebook group helps them cope, participants most commonly stated they *“feel like they help other families and other families help them”*, and they were *“more acceptant of reality”*. No respondent stated they were *“less hopeful for the future”* and only one parent stated their *“stress levels were higher”*. Ninety‐six percent (26/27) of parents stated they would like FIRES research advertised on social media, and only one parent stated they had no opinion on the matter (Table 2).

### 3.4 Recommendations from FIRES parents

**FIGURE 3:**
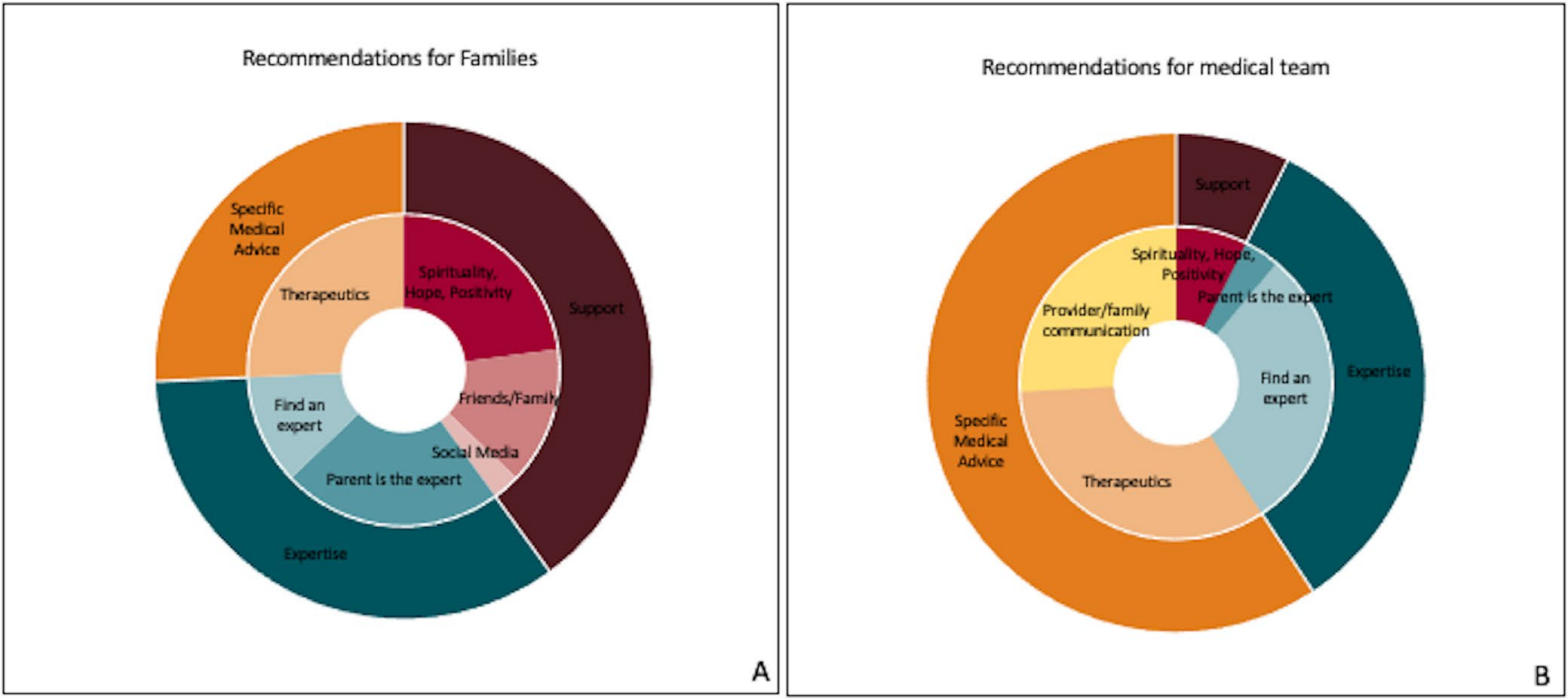
A total of 62 commentaries were shared by 25 parents. Thematic distribution of recommendations to families (A) and to medical team (B). For families, commentaries included the following themes: (1) Support: Spirituality/Hope/Positivity (n = 8), Friends/Family (n = 5), Social media (n = 1), (2) Expertise: empowering parents to become experts (n = 8), finding experts (n = 4), and (3) Specific medical advice: therapeutics (n = 9). For the medical team thematic distribution included: (1) Support: Spirituality/Hope/Positivity (n = 2), (2) Expertise: empowering parents to become experts (n = 1), finding experts (n = 8), and (3) Specific medical advice: therapeutics (n = 9), provider/family communication tips (n = 7)

Twenty‐five parents shared their recommendations in an open‐ended format. The themes included (1) support (spiritual, human, social media), (2) expertise (find experts, empower families to become experts), and (3) specific medical advice (therapeutics, medical team/family communication tips). Recommendations for fellow parents included 14 commentaries of support, 12 of expertise, and 9 of medical advice. For the medical team, 15 commentaries were made regarding specific medical advice, 7 regarding support, and 2 regarding expertise. In addition, 4 families expressed gratitude to the medical team as recommendations (Figure 3).

## 4 DISCUSSION

To our knowledge, this is the first parent‐reported assessment of FIRES, and it is outcomes, parental emotional states, and social media usage. Our cohort represented mothers more than fathers. The children affected were predominantly male, presented with FIRES at a median age of 5.6 years, and at time of the survey had a median age of 10.4. Two children died. Survivors experienced static poor functional outcomes and high rates of medically refractory epilepsy. Self‐reported parental depression, anxiety, and stress symptoms were relatively few. Parents expressed positive views on the social media group with regard to coping and their desire to have more research advertised. In an open‐ended format, they shared valuable recommendations for other families and for the medical community.

While the purpose of this publication was not to perform a retrospective analysis of characteristics of patients with FIRES, we chose to compare our cohort to other published reports to ensure validity of our sample and adequately interpret results. Comparing with another series of 29 children,^12^ the children in our series were younger, more often male, and included fewer deceased children. The children had similar rates and duration of intubation, lower rate of return to normal neurologic function, and they required more anti‐seizure medicines. Comparing with another cohort,^13^ our series included more males, younger age, fewer deaths, higher rates of prodromal gastrointestinal illness, similar hospitalization length, slightly lower anesthetic infusion duration, more anti‐seizure medicines, and worse outcomes.

By the nature of our recruitment strategy, it was logical that our sample would include fewer parents of children who died from FIRES or the converse, those who had gone back to their pre‐morbid baseline. When analyzing outcomes for the children in our cohort, it was clear that they were on the more severe spectrum of disability. An interesting aspect of their outcome was the relative static nature of their disability perceived by their parent, as 73% had no reported improvement in functional state across time. There are publications addressing prognostic factors after pediatric status epilepticus^14^, ^15^, ^16^, ^17^; however, data specific to NORSE and its subcategory FIRES are non‐existent to date. More so, to the best of our knowledge, there exists no literature that studies outcome variations for FIRES longitudinally. This is a potential area of research that may assist clinicians with better counseling of families.

Our cohort included parents of children with significant medical challenges, including medically refractory epilepsy in all survivors. Parents of children with epilepsy, particularly mothers, are at risk for depression, anxiety, and stress^18^ with significant disruption in their quality of life.^19^ Considering the limitation of a small sample size, we did not find this in our study. The rates of all mental health symptoms were low. We chose to analyze variables associated with symptoms of mental health distress solely among parents of survivors, as we hypothesized many variables may relate to the chronic FIRES period (such as number of seizures, anti‐seizure drugs, nursing assistance) It is worth noting that the parents of the two children who died reported symptoms of depression, anxiety, and stress in the normal range. Overall, the only variable correlating with depression was a higher number of weekly seizures which could translate into case severity and poorer outcomes. Further studies analyzing emotional states and mental health in parents of children with NORSE and its subcategory FIRES, as well as associated variables, may direct more targeted support.

Social support is a key factor in coping. Social media has become a ubiquitous way of socializing and among parents of sick children can assist with coping and supportive exchanges. Studies have also indicated social media can assist in parental support during the process of grief.^20^ We hypothesize this group of parents found the FIRES Facebook group beneficial regarding coping with their child's illness and thus reported fewer symptoms of depression, anxiety, and stress. These data are to be examined with caution, as there is no publication that addresses the negative emotional side effects of social media usage among parents of children with chronic illness, in addition to other detriments which may include misinformation and biases (reporting, sampling, and recall).^21^

Another benefit of social media involvement is the enhancement of research participation.^22^ This is especially crucial in rare diseases such as FIRES. In this cohort, advertising research on their social media platform was a welcome prospect. There are several aspects unique to this attractive form of recruiting such as website policies, investigator transparency, ensuring privacy, and managing online communication with participants. There exist guidelines available to ensure ethical considerations are incorporated when preparing a protocol that aims to use social media as a recruitment tool.^23^ Future FIRES research may include the consideration of recruitment strategies that utilize social media platforms.

We obtained valuable advice from families in an open‐ended format. We noticed a thematic difference in the recommendations made to fellow families compared with those made to the medical team. While some differences were predictable such as recommendations of seeking support (14 commentaries to families vs 2 commentaries to the medical team) and communication between medical team and families (7 commentaries to medical team 0 to families), some were less so. There was a high proportion of commentaries in the theme of expertise directed at empowering families to become experts in their child's illness versus finding that expertise elsewhere (8 vs 4). Conversely, all but 1 of the commentaries for the medical team regarding expertise encouraged them to seek experts if they lacked experience with this condition.

Parents of chronically ill children utilize social media apps, like Facebook, to exchange health‐related information.^24^ Many recommendations made by this cohort included highly specialized medical content that was mostly accurate and consistent with medical literature. For example, seven parents recognized the superiority of ketogenic diet over other anti‐seizure regimens and recommended its early initiation.^2^, ^25^ Five parents shared concerns of pentobarbital, and continuous infusion of midazolam to induce therapeutic coma was an independent factor associated with poorer outcomes.^26^, ^27^, ^28^ One parent stated similarities between phenobarbital and pentobarbital and questioned whether higher levels of phenobarbital could be permitted.^29^, ^30^ Two parents stated that a few seizures are expected and could be tolerated during the weaning process of continuous infusions.^31^ The educational background of families was not inquired, and thus, it is unknown whether they had pre‐existent medical education in any form or whether this is information acquired after their children became ill.

Our publication has several limitations. First, despite best attempts in social media visibility, we were only able to gather data on a small number of patients suffering from a very rare condition. Second, it is a survey‐based study and thus subject to bias. This is especially relevant for sensitive data like depression, anxiety, and stress levels which is subject to reporting bias and thus generalizable conclusions about parental mental health are unable to be made, especially in a small cohort. At the time of survey completion, all children were in the chronic phase of FIRES and thus the details of the acute period may not be recent, thus making the data subject to recall bias. However, parents indicated they took detailed notes and likely referred to them when answering the survey as we have gathered very granular data (ie, duration of medically induced coma, dates of intubation, complications). This cohort is subject to selection bias as the recruitment was performed via social media. Those who participated are active in the FIRES group making their assessment of social media impact in coping possibly more positive, as those who did not find it efficacious most likely left the platform or are not active. Third, this study intends to describe the FIRES journey through the parent perspective and thus specific medical data may be subject to inaccuracies. Lastly, and importantly, this study provides a static assessment of an otherwise dynamic condition.

## 5 CONCLUSION

Febrile infection‐related epilepsy syndrome has devastating consequences that impact caregivers as well as patients and are not isolated to the acute period. There remain many unanswered questions pertaining to FIRES including early biomarkers, etiology, ideal therapeutics, outcomes, and neuro‐prognostication. Research must also focus on understanding and supporting the family caring for the child and enhancing communication between the medical team and the parents. But given its rarity, creativity is necessary to involve a larger number of patients over a wider spectrum of phases and themes. This study highlights the much‐needed partnership between clinicians, scientists, and families of children with rare diseases such as FIRES and explores social media as a welcome introductory venue to understand complex family issues and enhance the clinician/family relationship.

## DISCLOSURES

None of the authors have any conflicts of interest to disclose. We confirm that we have read the Journal's position on issues involved in ethical publication and affirm that this report is consistent with those guidelines.

## Supporting information

Appendix S1
