# Supplementary material for: Parental perception of FIRES outcomes, emotional states, and social media usage
Source: Epilepsia Open. 2021 Jun 21;6(3):539–47. doi: 10.1002/epi4.12513 (PMC8408589; doi:10.1002/epi4.12513)
Supplement: Supplementary file 1 — Appendix S1 [file EPI4-6-539-s001.docx]

FIRES SURVEY

Start of Block: Initial questions

Q1.1
**SCREENING QUESTIONS**

Q1.2 Did your child have refractory status epilepticus (a really long seizure state needing several seizure drugs) without a known cause?

- Yes (1)
- No (2)

Skip To: Q1.6 If Did your child have refractory status epilepticus (a really long seizure state needing several se... = No

Q1.3 Prior to the status epilepticus was your child healthy overall, with relatively typical development and without epilepsy? 

- Yes (1)
- No (2)

Q1.4 Did your child have a fever 1 to 14 days prior to status epilepticus onset?

- Yes (1)
- No (2)

Display This Question:

If Did your child have a fever 1 to 14 days prior to status epilepticus onset? = Yes

Q1.5 Please describe the illness associated with the fever.
Check all that apply:

- Gastrointestinal (vomiting, diarrhea, abdominal pain) (1)
- Upper respiratory (runny nose, cough, congestion, sneezing, sore throat) (2)
- Rash (3)
- Lower respiratory (cough, heavy breathing, fatigue during activity) (4)
- Other (5) ________________________________________________

| 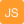 |
| --- |

Q1.6 What is your child's date of birth?

|  |  |
| --- | --- |
| Month (1) | ▼ January (1) ...   (150) |
| Day (2) | ▼ January (1) ...   (150) |
| Year (3) | ▼ January (1) ...   (150) |

Q1.7 What is your child's gender?

- Male (1)
- Female (2)

Q1.8 In which country did your child develop FIRES?

- Country (1) ________________________________________________

End of Block: Initial questions

Start of Block: Pre FIRES presentation section

Q2.1
**PRE FIRES PRESENTATION**

Q2.2 Please state your relationship to the child with FIRES.

- Mother (1)
- Father (2)
- Grandmother (3)
- Grandfather (4)
- Other (5) ________________________________________________

Q2.3 Did your child have a medical history of epilepsy (i.e. seizure disorder) prior to their first seizure that led to FIRES?

- Yes (1)
- No (2)

Q2.4 Did your child have a history of febrile seizures? (i.e. seizures in children 6 months to 6 years provoked by fevers with return to normal after the seizure).

- Yes (1)
- No (2)

End of Block: Pre FIRES presentation section

Start of Block: Acute FIRES presentation section

Q3.1     **ACUTE FIRES PRESENTATION**   The acute FIRES stage is defined as the time when your child first developed refractory status epilepticus (really long seizures requiring multiple drugs to control seizures, often a continuous drip of anesthesia is needed for seizure control).

| 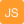 |
| --- |

Q132 When did your child first present with status epilepticus leading to FIRES?

|  |  |
| --- | --- |
| Month (1) | ▼ January (1) ...   (150) |
| Day (2) | ▼ January (1) ...   (150) |
| Year (3) | ▼ January (1) ...   (150) |
| Do not remember (4) | ▼ January (1) ...   (150) |

Q3.3
Was your child ever in a medically induced coma (needed a continuous infusion drug to control seizures and maintain a very deep sedated state) in the acute FIRES stage?

- Yes (2)
- No (3)
- I do not remember / Unknown / Prefer not to answer (1)

Display This Question:

If Was your child ever in a medically induced coma (needed a continuous infusion drug to control sei... = Yes

Q3.4
How many days was your child in a medically induced coma?

- Days (1) ________________________________________________
- I do not remember / Unknown / Prefer not to answer (2)

Q3.5
How many days did it take from first presentation with status epilepticus until your child was diagnosed with FIRES?

- Days (1) ________________________________________________
- I do not remember / Unknown / Prefer not to answer (2)

Q3.6 Who first diagnosed your child with FIRES?

- Neurologist/Epileptologist in the hospital (1)
- Neurologist/Epileptologist in the clinic (2)
- Intensivist in the ICU (3)
- Infectious disease specialist (4)
- Rheumatologist (5)
- General pediatrician (6)
- Other (7) ________________________________________________
- I do not remember / Unknown / Prefer not to answer (8)

Q3.7 What would you recommend to a family in a similar situation?

________________________________________________________________

________________________________________________________________

________________________________________________________________

________________________________________________________________

________________________________________________________________

Q3.8 What would be your recommendation for the hospital team?

________________________________________________________________

________________________________________________________________

________________________________________________________________

________________________________________________________________

________________________________________________________________

End of Block: Acute FIRES presentation section

Start of Block: Acute FIRES complications section

Q4.1 **ACUTE FIRES COMPLICATIONS**
 In the Acute FIRES stage (when your child was admitted with refractory status epilepticus for the first time):

Q4.2 Was / Is your child admitted to a hospital?

- Yes, my child was / is admitted to a hospital (1)
- No, my child was / is not admitted to a hospital (2)
- I do not remember / Unknown / Prefer not to answer (3)

Display This Question:

If Was / Is your child admitted to a hospital? = Yes, my child was / is admitted to a hospital

Q4.3 What area of the hospital was / is your child admitted to?

- Regular floor (1)
- Intensive care unit (2)
- Both (3)
- I do not remember / Unknown / Prefer not to answer (4)

Display This Question:

If Was / Is your child admitted to a hospital? = Yes, my child was / is admitted to a hospital

Q4.4
What was/is the duration of your child's stay?


Please indicate the amount of days.

- Currently in the regular floor or ICU: (1) ________________________________________________
- Was in regular floor or ICU: (2) ________________________________________________
- I do not remember / Unknown / Prefer not to answer (3)

Display This Question:

If Was / Is your child admitted to a hospital? = Yes, my child was / is admitted to a hospital

Q4.5
Does / Did your child have a breathing tube in their mouth?


If yes, indicate how many days the tube has been / was in. Leave box blank if you're unsure the amount of days.

- Yes (1) ________________________________________________
- No (2)
- I do not remember / Unknown / Prefer not to answer (3)

Q4.6
Did your child have any of the following complications in the acute FIRES stage?


Check all that apply:

- Sepsis (i.e. an extreme response to infection with inflammation of the body which can cause damage to other organs) (1)
- Deep vein thrombosis (i.e blood clot) needing blood thinners (2)
- Cardiac arrest (i.e. heart stopped and they needed to do CPR) (3)
- Intestinal (i.e. bowel) problem like severe diarrhea, constipation, bowel perforation, or bowel necrosis (i.e. bowel death) (4)
- Multiple organs (i.e. kidneys, liver, bone marrow, lungs, etc.) fail (5)
- Skin rash (6)
- Dysautonomia or neurostorming (i.e. very high blood pressure, heart rate, temperature, breathing rate, large pupils, sweating, abnormal movement or tremors that were not seizures) (7)
- I do not remember / Unknown / Prefer not to answer (8)

End of Block: Acute FIRES complications section

Start of Block: Sub acute/Chronic FIRES section & coping

Q5.1
**SUB-ACUTE FIRES**
 In the sub-acute stage, the patient may remain in the hospital or is being discharged from the hospital. The seizures are either well controlled or treated less aggressively.

 If your child is still in the acute period or did not survive the acute period, please select "Skip" to be directed to the parent coping section; otherwise, please choose "Sub-acute" to continue.

- Skip (1)
- Sub-acute (2)

Skip To: Q5.45 If SUB-ACUTE FIRES  In the sub-acute stage, the patient may remain in the hospital or is being disch... = Skip

Q5.2
Did your child need a tracheostomy (breathing tube directly in the throat)?

- Yes (1)
- No (2)
- Unknown (3)

Display This Question:

If Did your child need a tracheostomy (breathing tube directly in the throat)? = Yes

| 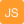 |
| --- |

Q5.3 When was the tracheostomy placed?

|  |  |
| --- | --- |
| Month (1) | ▼ January (1) ...   (150) |
| Day (2) | ▼ January (1) ...   (150) |
| Year (3) | ▼ January (1) ...   (150) |
| Do not remember (4) | ▼ January (1) ...   (150) |

Q5.4
Did your child need a gastrostomy to feed (feeding tube directly in the stomach)?

- Yes (1)
- No (2)
- Unknown (3)

Display This Question:

If Did your child need a gastrostomy to feed (feeding tube directly in the stomach)?  = Yes

| 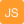 |
| --- |

Q5.5 When was the gastrostomy placed?

|  |  |
| --- | --- |
| Month (1) | ▼ January (1) ...   (150) |
| Day (2) | ▼ January (1) ...   (150) |
| Year (3) | ▼ January (1) ...   (150) |
| Do not remember (4) | ▼ January (1) ...   (150) |

Q5.6 Has your child been discharged from the hospital?

- Yes (1)
- No (2)

Skip To: Q5.45 If Has your child been discharged from the hospital? = No

| 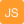 |
| --- |

Q5.7 When was your child discharged from the hospital?

|  |  |
| --- | --- |
| Month (1) | ▼ January (1) ...   (150) |
| Day (2) | ▼ January (1) ...   (150) |
| Year (3) | ▼ January (1) ...   (150) |
| Do not remember (4) | ▼ January (1) ...   (150) |

Q5.8 Where was your child discharged to?

- Rehabilitation (1)
- Sub-acute care facility (2)
- Home (3)
- Other (4) ________________________________________________
- Unknown (5)

Skip To: Q5.10 If Where was your child discharged to? = Home

Skip To: Q5.10 If Where was your child discharged to? = Unknown

Q5.9 If your child went to rehabilitation or another facility after discharge please check one of the following and indicate the amount of days:

- Currently in rehabilitation/other facility: (1) ________________________________________________
- Was in rehabilitation/other facility: (2) ________________________________________________
- Was in rehabilitation/other facility but I don't remember for how long. (3)
- Was never in rehabilitation/other facility. (4)
- Died while in rehabilitation/other facility. (5)
- Unknown (6)

Q5.10 **At discharge from the hospital after acute FIRES**, please indicate how your child was functioning according to the following scale:

Q5.11
**1) Consciousness:**
1a) Is your child able to obey simple commands or say any words? Or for younger patients: can your child act / react / interact beyond reflexes?

 An individual who shows the ability to obey even simple commands or utter any word or communicate specifically in any other way is not considered to be in a vegetative state. Eye movements are not reliable evidence of meaningful responsiveness. However, for infants, actively following the movement of a parent or people/object with eyes, grasping for objects, making faces, etc. are interactions, breast feeding and crying continuously can be reflexes.

- Yes (1)
- No (2)

Skip To: Q5.23 If 1) Consciousness: 1a) Is your child able to obey simple commands or say any words? Or for younger... = No

Q5.12
**2) Independence in the home:**

2a) Is the assistance of another person at home essential every day for some activities of daily living? Or for younger patients: is the child dependent upon a caregiver more so than is expected based on age?


For an older child, complete independence and a 'no' answer should mean the child can get washed, put on clean clothes without prompting, prepare food for themselves, deal with callers, and handle minor domestic crises. The child should be able to carry out activities without needing prompting or reminding and should be capable of being left alone for an age appropriate period. Young children should be able to accomplish age appropriate developmental milestones without assistance.

- Yes (1)
- No (2)

Skip To: Q5.14 If 2) Independence in the home: 2a) Is the assistance of another person at home essential every day... = No

Q5.13 2b) Does the child need frequent help or for someone to be around at home most of the time? Or for younger patients: does the child need frequent help from a caregiver to accomplish tasks that a child this age should be able to accomplish (if child sometimes functions at an age appropriate level, then answer 'no').

- Yes (1)
- No (2)

Skip To: Q5.23 If 2b) Does the child need frequent help or for someone to be around at home most of the time? Or fo... = Yes

Skip To: Q5.23 If 2b) Does the child need frequent help or for someone to be around at home most of the time? Or fo... = No

Q5.14
**3) Independence outside the home:**
3a) Is the child able to shop and travel without assistance? Or for younger patients: does the child behave age apprpriately outside the home?


This item considers activities such as shopping and traveling in the context of age appropriate behaviors. This includes being able to plan what to buy, take care of money, and behave appropriately in public. The individual need not normally shop, but must be capable of doing so. A younger child must behave age appropriately in public. An older child may drive or use public transit to get around. Ability to use a taxi is sufficient, provided the person can phone for it themselves and give instructions to the driver. Older children who sometimes were allowed to travel independently before the injury should be able to walk to a neighbor's house, take a school bus, ride a bike, or take public transportation.

- Yes (1)
- No (2)

Skip To: Q5.23 If 3) Independence outside the home: 3a) Is the child able to shop and travel without assistance? Or... = No

Q5.15
**4) School/Work:**
4a) Can the child function at work or in school at his or her previous capacity?


If an adolescent was working before the injury, then his or her current capacity for work should be at the same level. If the individual was seeking work before, then the injury should not have adversely affected chances of obtaining work or the level of work for which he or she is eligible. If the patient was in preschool or a student before the injury, then capacity for school work and school activities should not be adversely affected. 

- Yes (1)
- No (2)

Skip To: Q5.18 If 4) School/Work: 4a) Can the child function at work or in school at his or her previous capacity?... = Yes

Q5.16
**4b) Level of restriction:**
i) Able to work in a sheltered workshop or non-cometetive job, in a school setting for severely impaired children or tutored at home, or currently unable to work or go to school.

- Yes (1)
- No (2)

Skip To: Q5.23 If 4b) Level of restriction: Able to work in a sheltered workshop or non-cometetive job, in a school se = Yes

Q5.17 ii) Does your child have a reduced work or school capacity?

- Yes (1)
- No (2)

Skip To: Q5.23 If ii) Does your child have a reduced work or school capacity? = Yes

Q5.18
**5) Social & Leisure Activities:**
5a) Is the child able to resume regular social and leisure activities?


The individual may not have resumed all previous leisure activities but should not be prevented from doing so by physical or mental impairment. If he or she has stopped the majority of activities because of loss of interest or motivation, then this is considered a disability. For younger children, social and leisure activities can include games and toys played with caretakers, siblings or other children, as well as the ability to interact in a playful manner with others.

- Yes (1)
- No (2)

Skip To: Q5.20 If 5) Social & Leisure Activities: 5a) Is the child able to resume regular social and leisure activi... = Yes

Q5.19
5b) What is the extent of restrictions on social and leisure activities?

- i) Unable to participate: rarely, if ever, take part. (1)
- ii) Participate much less: less than half as often (2)
- iii) Participate a bit less: at least half as often as before injury. (3)

Skip To: Q5.23 If 5b) What is the extent of restrictions on social and leisure activities?   = i) Unable to participate: rarely, if ever, take part.

Skip To: Q5.23 If 5b) What is the extent of restrictions on social and leisure activities?   = iii) Participate a bit less: at least half as often as before injury.

Skip To: Q5.23 If 5b) What is the extent of restrictions on social and leisure activities?   = ii) Participate much less: less than half as often

Q5.20
**6) Family & Friends:**
 6a) Are there psychological problems that have resulted in ongoing disruption with respect to either family or friendships?


Quick temper, irritability, anxiety, aggressive acts, insensitivity to others, mood swings, depression, and unreasonable or childish behavior that is not age appropriate. 

- Yes (1)
- No (2)

Skip To: Q5.22 If 6) Family & Friends: 6a) Are there psychological problems that have resulted in ongoing disruptio... = No

Q5.21 6b) What is the extent of disruption or strain?

- Constant - daily and intolerable (1)
- Frequent - once a week, but more tolerable (2)
- Occasional - less than weekly (3)

Skip To: Q5.23 If 6b) What is the extent of disruption or strain? = Constant - daily and intolerable

Skip To: Q5.23 If 6b) What is the extent of disruption or strain? = Occasional - less than weekly

Skip To: Q5.23 If 6b) What is the extent of disruption or strain? = Frequent - once a week, but more tolerable

Q5.22
**7) Return to normal life:**
 7a) Are there any other problems relating the injury that affect daily life?


Headaches, dizziness, tiredness, sensitivity to noise or light, slowness, memory failures, concentration problems, or other problems.

- Yes (1)
- No (2)

Q5.23
**CHRONIC FIRES**
In the chronic stage, the child has typically returned home.

 If your child is still in the acute or sub-acute period, or did not survive the acute or sub-acute period please choose "Skip" to be directed to the parent coping section; otherwise, please choose "Chronic FIRES" to continue.

- Skip (1)
- Chronic FIRES (2)

Skip To: Q5.45 If CHRONIC FIRES In the chronic stage, the child has typically returned home. If your child is still... = Skip

Q5.24 How many seizures does your child have per week?

________________________________________________________________

Q5.25 How many seizure medications does your child take daily?

________________________________________________________________

Q5.26 Does your child qualify for public insurance and/or long-term governmental support?

- Yes (1)
- No (2)
- Unknown (3)

Q5.27 Does your child receive nursing support at home?

- Yes (1)
- No (2)

Q5.28 Does your child receive therapies?

- Yes (1)
- No (2)

Display This Question:

If Does your child receive therapies? = Yes

Q5.29
What type of therapy does your child receive?


Check all that apply and indicate hours per week:

- Occupational (1) ________________________________________________
- Physical (2) ________________________________________________
- Speech (3) ________________________________________________
- Other (4) ________________________________________________

Q5.30 Who does the primary medical management for your child's chronic FIRES?

- Neurologist/Epileptologist in the clinic (1)
- Rheumatologist (2)
- General pediatrician (3)
- Other (4) ________________________________________________
- Unknown (5)

Q5.31 How many neurologists has your child seen since first diagnosed?

________________________________________________________________

Q5.32 Please indicate how your child **functions currently in the chronic stage of FIRES** according to the following scale:

Q5.33

**1) Consciousness:**
1a) Is your child able to obey simple commands or say any words? Or for younger patients: can your child act / react / interact beyond reflexes?
 
An individual who shows the ability to obey even simple commands or utter any word or communicate specifically in any other way is not considered to be in a vegetative state. Eye movements are not reliable evidence of meaningful responsiveness. However, for infants, actively following the movement of a parent or people/object with eyes, grasping for objects, making faces, etc. are interactions, breast feeding and crying continuously can be reflexes.

- Yes (1)
- No (2)

Skip To: Q5.45 If 1) Consciousness: 1a) Is your child able to obey simple commands or say any words? Or for younger... = No

Q5.34


**2) Independence in the home:**

2a) Is the assistance of another person at home essential every day for some activities of daily living? Or for younger patients: is the child dependent upon a caregiver more so than is expected based on age?
 
For an older child, complete independence and a 'no' answer should mean the child can get washed, put on clean clothes without prompting, prepare food for themselves, deal with callers, and handle minor domestic crises. The child should be able to carry out activities without needing prompting or reminding and should be capable of being left alone for an age appropriate period. Young children should be able to accomplish age appropriate developmental milestones without assistance.

- Yes (1)
- No (2)

Skip To: Q5.36 If 2) Independence in the home: 2a) Is the assistance of another person at home essential every day... = No

Q5.35 2b) Does the child need frequent help or for someone to be around at home most of the time? Or for younger patients: does the child need frequent help from a caregiver to accomplish tasks that a child this age should be able to accomplish (if child sometimes functions at an age appropriate level, then answer 'no').

- Yes (1)
- No (2)

Skip To: Q5.45 If 2b) Does the child need frequent help or for someone to be around at home most of the time? Or fo... = Yes

Skip To: Q5.45 If 2b) Does the child need frequent help or for someone to be around at home most of the time? Or fo... = No

Q5.36


**3) Independence outside the home:**
3a) Is the child able to shop and travel without assistance? Or for younger patients: does the child behave age apprpriately outside the home?


This item considers activities such as shopping and traveling in the context of age appropriate behaviors. This includes being able to plan what to buy, take care of money, and behave appropriately in public. The individual need not normally shop, but must be capable of doing so. A younger child must behave age appropriately in public. An older child may drive or use public transit to get around. Ability to use a taxi is sufficient, provided the person can phone for it themselves and give instructions to the driver. Older children who sometimes were allowed to travel independently before the injury should be able to walk to a neighbor's house, take a school bus, ride a bike, or take public transportation.

- Yes (1)
- No (2)

Skip To: Q5.45 If 3) Independence outside the home: 3a) Is the child able to shop and travel without assistance? Or... = No

Q5.37

**4) School/Work:**
4a) Can the child function at work or in school at his or her previous capacity?


If an adolescent was working before the injury, then his or her current capacity for work should be at the
same level. If the individual was seeking work before, then the injury should not have adversely affected chances of obtaining work or the level of work for which he or she is eligible. If the patient was in preschool or a student before the injury, then capacity for school work and school activities should not be adversely affected.

- Yes (1)
- No (2)

Display This Question:

If 4) School/Work: 4a) Can the child function at work or in school at his or her previous capacity?... = No

Q5.38

**4b) Level of restriction:**
i) Able to work in a sheltered workshop or non-cometetive job, in a school setting for severely impaired children or tutored at home, or currently unable to work or go to school.

- Yes (1)
- No (2)

Skip To: Q5.45 If 4b) Level of restriction: i) Able to work in a sheltered workshop or non-cometetive job, in a sch... = Yes

Display This Question:

If 4b) Level of restriction: i) Able to work in a sheltered workshop or non-cometetive job, in a sch... = No

Q5.39 ii) Does your child have a reduced work or school capacity?

- Yes (1)
- No (2)

Skip To: Q5.45 If ii) Does your child have a reduced work or school capacity? = Yes

Q5.40


**5) Social & Leisure Activities:**
5a) Is the child able to resume regular social and leisure activities?


The individual may not have resumed all previous leisure activities but should not be prevented from doing so by physical or mental impairment. If he or she has stopped the majority of activities because of loss of interest or motivation, then this is considered a disability. For younger children, social and leisure activities can include games and toys played with caretakers, siblings or other children, as well as the ability to interact in a playful manner with others.

- Yes (1)
- No (2)

Display This Question:

If 5) Social & Leisure Activities: 5a) Is the child able to resume regular social and leisure activi... = No

Q5.41
5b) What is the extent of restrictions on social and leisure activities?

- i) Unable to participate: rarely, if ever, take part. (1)
- ii) Participate much less: less than half as often (2)
- iii) Participate a bit less: at least half as often as before injury. (3)

Skip To: Q5.45 If 5b) What is the extent of restrictions on social and leisure activities?   = i) Unable to participate: rarely, if ever, take part.

Skip To: Q5.45 If 5b) What is the extent of restrictions on social and leisure activities?   = ii) Participate much less: less than half as often

Skip To: Q5.45 If 5b) What is the extent of restrictions on social and leisure activities?   = iii) Participate a bit less: at least half as often as before injury.

Q5.42
**6) Family & Friends:** 6a) Are there psychological problems that have resulted in ongoing disruption with respect to either family or friendships?


Quick temper, irritability, anxiety, aggressive acts, insensitivity to others, mood swings, depression, and unreasonable or childish behavior that is not age appropriate. 

- Yes (1)
- No (2)

Display This Question:

If 6) Family & Friends: 6a) Are there psychological problems that have resulted in ongoing disruptio... = Yes

Q5.43 6b) What is the extent of disruption or strain?

- Constant - daily and intolerable (1)
- Frequent - once a week, but more tolerable (2)
- Occasional - less than weekly (3)

Q5.44

**7) Return to normal life:**
 7a) Are there any other problems relating the injury that affect daily life?


Headaches, dizziness, tiredness, sensitivity to noise or light, slowness, memory failures, concentration problems, or other problems.

- Yes (1)
- No (2)

Q5.45
**PARENT COPING**
Please read each statement and choose a number 0, 1, 2, or 3 according to how much the statement applied to you over the past week. Do not spend too much time on any statement.

 The rating scale is as follows:
 0 Did not apply to me at all
 1 Applied to me to some degree, or some of the time
 2 Applied to me to a considerable degree, or a good part of the time
 3 Applied to me very much, or most of the time

Q5.46 I could not see anything in the future to be hopeful about

- 0 (1)
- 1 (2)
- 2 (3)
- 3 (4)

Q5.47 I couldn't seem to experience any positive feeling at all

- 0 (1)
- 1 (2)
- 2 (3)
- 3 (4)

Q5.48 I couldn't seem to get any enjoyment out of the things I did

- 0 (1)
- 1 (2)
- 2 (3)
- 3 (4)

Q5.49 I experienced breathing difficulty (i.e. excessively rapid breathing, breathlessness in the absence of physical exertion)

- 0 (1)
- 1 (2)
- 2 (3)
- 3 (4)

Q5.50 I experienced trembling (i.e. in the hands)

- 0 (1)
- 1 (2)
- 2 (3)
- 3 (4)

Q5.51 I feared that I would be "thrown" by some trivial but unfamiliar task

- 0 (1)
- 1 (2)
- 2 (3)
- 3 (4)

Q5.52 I felt down-hearted and blue

- 0 (1)
- 1 (2)
- 2 (3)
- 3 (4)

Q5.53 I felt I was pretty worthless

- 0 (1)
- 1 (2)
- 2 (3)
- 3 (4)

Q5.54
I felt I wasn't worth much as a person

- 0 (1)
- 1 (2)
- 2 (3)
- 3 (4)

Q5.55 I felt sad and depressed

- 0 (1)
- 1 (2)
- 2 (3)
- 3 (4)

Q5.56 I felt scared without any good reason

- 0 (1)
- 1 (2)
- 2 (3)
- 3 (4)

Q5.57 I felt terrified

- 0 (1)
- 1 (2)
- 2 (3)
- 3 (4)

Q5.58 I felt that I had lost interest in just about everything

- 0 (1)
- 1 (2)
- 2 (3)
- 3 (4)

Q5.59 I felt that I had nothing to look forward to

- 0 (1)
- 1 (2)
- 2 (3)
- 3 (4)

Q5.60 I felt that I was close to panic

- 0 (1)
- 1 (2)
- 2 (3)
- 3 (4)

Q5.61 I felt that I was rather touchy

- 0 (1)
- 1 (2)
- 2 (3)
- 3 (4)

Q5.62 I felt that I was using a lot of nervous energy

- 0 (1)
- 1 (2)
- 2 (3)
- 3 (4)

Q5.63 I felt that life was meaningless

- 0 (1)
- 1 (2)
- 2 (3)
- 3 (4)

Q5.64 I felt that life wasn't worthwhile

- 0 (1)
- 1 (2)
- 2 (3)
- 3 (4)

Q5.65 I found it difficult to relax

- 0 (1)
- 1 (2)
- 2 (3)
- 3 (4)

Q5.66 I found it difficult to tolerate interruption to what I was doing

- 0 (1)
- 1 (2)
- 2 (3)
- 3 (4)

Q5.67 I found it difficult to work up the initiative to do things

- 0 (1)
- 1 (2)
- 2 (3)
- 3 (4)

Q5.68 I found it hard to calm down after something upset me

- 0 (1)
- 1 (2)
- 2 (3)
- 3 (4)

Q5.69 I found it hard to wind down

- 0 (1)
- 1 (2)
- 2 (3)
- 3 (4)

Q5.70 I found myself getting agitated

- 0 (1)
- 1 (2)
- 2 (3)
- 3 (4)

Q5.71 I found myself getting impatient when I was delayed in any way (i.e. elevators, traffic lights, being kept waiting)

- 0 (1)
- 1 (2)
- 2 (3)
- 3 (4)

Q5.72 I found myself getting upset by quite trivial things

- 0 (1)
- 1 (2)
- 2 (3)
- 3 (4)

Q5.73 I found myself getting upset rather easily

- 0 (1)
- 1 (2)
- 2 (3)
- 3 (4)

Q5.74 I found myself in situations that made me so anxious I was most relieved when they ended

- 0 (1)
- 1 (2)
- 2 (3)
- 3 (4)

Q5.75 I found that I was very irritable

- 0 (1)
- 1 (2)
- 2 (3)
- 3 (4)

Q5.76 I had a feeling of faintness

- 0 (1)
- 1 (2)
- 2 (3)
- 3 (4)

Q5.77 I had a feeling of shakiness (i.e. legs going to give way)

- 0 (1)
- 1 (2)
- 2 (3)
- 3 (4)

Q5.78 I had difficulty in swallowing

- 0 (1)
- 1 (2)
- 2 (3)
- 3 (4)

Q5.79 I just couldn't seem to get going

- 0 (1)
- 1 (2)
- 2 (3)
- 3 (4)

Q5.80 I perspired noticeably (i.e. hands sweaty) in the absence of high temperatures or physical exertion

- 0 (1)
- 1 (2)
- 2 (3)
- 3 (4)

Q5.81 I tended to over-react to situations

- 0 (1)
- 1 (2)
- 2 (3)
- 3 (4)

Q5.82 I was aware of dryness of my mouth

- 0 (1)
- 1 (2)
- 2 (3)
- 3 (4)

Q5.83 I was aware of the action of my heart in the absence of physical exertion (i.e. sense of heart rate increase, heart missing a beat)

- 0 (1)
- 1 (2)
- 2 (3)
- 3 (4)

Q5.84 I was in a state of nervous tension

- 0 (1)
- 1 (2)
- 2 (3)
- 3 (4)

Q5.85 I was intolerant of anything that kept me from getting on with what I was doing

- 0 (1)
- 1 (2)
- 2 (3)
- 3 (4)

Q5.86 I was unable to become enthusiastic about anything

- 0 (1)
- 1 (2)
- 2 (3)
- 3 (4)

Q5.87 I was worried about situations in which I might panic and make a fool of myself

- 0 (1)
- 1 (2)
- 2 (3)
- 3 (4)

End of Block: Sub acute/Chronic FIRES section & coping

Start of Block: Social Media

Q6.1
**SOCIAL MEDIA**
 
Do you participate in social media with regard to FIRES?
 
Check all that apply:

- Facebook (1)
- Twitter (2)
- Instagram (3)
- Other (4) ________________________________________________
- No social media (5)
- I do not remember / Unknown / Prefer not to answer (6)

Q6.2 Do you participate in the F.I.R.E.S. Facebook group?

- Yes (1)
- No (2)
- I do not remember / Unknown / Prefer not to answer (3)

Skip To: Q6.15 If Do you participate in the F.I.R.E.S. Facebook group? = No

Skip To: Q6.15 If Do you participate in the F.I.R.E.S. Facebook group? = I do not remember / Unknown / Prefer not to answer

| 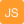 |
| --- |

Q6.3 When did you join the F.I.R.E.S. Facebook group?

|  |  |
| --- | --- |
| Month (1) | ▼ January (1) ...   (150) |
| Day (2) | ▼ January (1) ...   (150) |
| Year (3) | ▼ January (1) ...   (150) |
| Do not remember (4) | ▼ January (1) ...   (150) |

Q6.4 How has the F.I.R.E.S. Facebook group impacted you?

- Extremely positive (1)
- Moderately positive (2)
- Slightly positive (3)
- Neither positive nor negative (4)
- Slightly negative (5)
- Moderately negative (6)
- Extremely negative (7)
- I do not remember / Unknown / Prefer not to answer (8)

Q6.5
On a scale of 1-10 how has this group helped you with coping?


 1 = not helpful & 10 = most helpful

________________________________________________________________

Q6.6
On a scale of 1-10 how has this group helped you parent your child with FIRES?


1 = not helpful & 10 = most helpful

________________________________________________________________

Q6.7 Do you follow medical advice from the group?

- Yes (1)
- No (2)
- I do not remember / Unknown / Prefer not to answer (3)

Skip To: Q6.9 If Do you follow medical advice from the group? = No

Q6.8 If you follow medical advice from the group, do you:

- Check with your child's care team prior (1)
- Not check with your child's care team prior (2)
- I do not remember / Unknown / Prefer not to answer (3)

Q6.9 How many hours per week do you spend in this group?

________________________________________________________________

Q6.10 How many posts per week do you make in this group?

________________________________________________________________

Q6.11 Would you like your doctor / neurologist / epileptologist to be a member of the FIRES Facebook group?

- Yes (1)
- No (2)
- Unsure (3)

Q6.12
Has this group changed the way your child is cared for? 


Check all that apply:

- It has not changed (1)
- Started a new medication/diet (2)
- Discontinued a medication/diet (3)
- Redirected goals of care (4)
- Obtained a new test (5)
- Joined a research study (6)
- Changed physicians (7)
- Other (8) ________________________________________________

Q6.13
Has this group changed the way you cope with your child's disease?


Check all that apply:

- It has not changed how I cope (1)
- I've socialized in person with members (2)
- I feel like I help other families (3)
- I feel like other families help me (4)
- My stress levels are lower (5)
- My stress levels are higher (6)
- I was more hopeful for the future (7)
- I was less hopeful for the future (8)
- I was more acceptant of reality (9)
- Other (10) ________________________________________________
- I do not remember / Unknown / Prefer not to answer (11)

Q6.14 Would you like to see FIRES research advertised on social media?

- Yes (1)
- No (2)
- I don't have an opinion on this (3)
- I do not remember / Unknown / Prefer not to answer (4)

Q6.15 Did your child survive FIRES?

- Yes (1)
- No (2)
- I do not remember / Unknown / Prefer not to answer (3)

Display This Question:

If Did your child survive FIRES? = No

| 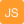 |
| --- |

Q133 What was your child's date of death?

|  |  |
| --- | --- |
| Month (1) | ▼ January (1) ...   (150) |
| Day (2) | ▼ January (1) ...   (150) |
| Year (3) | ▼ January (1) ...   (150) |

End of Block: Social Media
